# Synthesis and structure of hexa­aqua­cobalt bis­(2-methyl-1*H*-imidazol-3-ium) tetra­aqua­bis­(benzene-1,3,5-tri­carboxyl­ato-κ*O*)cobalt

**DOI:** 10.1107/S2056989022007046

**Published:** 2022-07-19

**Authors:** Jose de Jesus Velazquez-Garcia, Simone Techert

**Affiliations:** a Deutsches Elektronen-Synchrotron DESY, Notkestr. 85, 22607 Hamburg, Germany; b Institut für Röntgenphysik, Georg-August-Universität Göttingen, Friedrich-Hund-Platz 1, Göttingen, 37077, Germany; Universidad Nacional Autónoma de México, México

**Keywords:** crystal structure, hexa­aqua-cobalt, 2-methyl­imidazole, 1,3,5-benzene tri­carb­oxy­lic acid

## Abstract

The title compound, (C_4_H_7_N_2_)_2_[Co(H_2_O)_6_][Co(C_9_H_3_O_6_)_2_(H_2_O)_4_] (**1**), was synthesized from cobalt(II) chloride, 1,3,5-benzene tri­carb­oxy­lic acid (Hbtc) and 2-methyl-imidazole (H-2mIm) under ambient conditions. The structure of **1** is here reported and compared with the parent complex hexa­aqua­cobalt bis­(1*H*-imidazol-3-ium) tetra­aqua­bis­(benzene-1,3,5-tri­carboxyl­ato)cobalt (**2**).

## Abstract 2

An hexa­aqua­cobalt-based complex was synthesized and its structure determined by single-crystal X-ray diffraction. The observed Co—O_carboxyl­ate_ bond length is 2.0835 (9) Å and the Co—O_water_ bond lengths are in the range 2.0576 (9)-2.1196 (9) Å.

## 1. Chemical context

Effective bifunctional electrocatalysts for oxygen reduction/evolution reactions (ORR/OER) are indispensable for the development of energy storage and conversion systems, such as fuel cells and rechargeable metal–air batteries (Cai *et al.*, 2017 ▸; Wang *et al.*, 2014 ▸). Currently, platinum-based materials are considered the most effective due to their superior catalytic activity and stability. However, their high cost, caused by the scarcity of the metal, rules them out for scale-up development. Therefore, a great deal of effort has been devoted to the development of cost-effective and earth-abundant replacements for platinum-based catalysts. Among the different substitute materials, a hexa­aqua­cobalt bis­(1*H*-imidazol-3-ium) tetra­aqua­bis­(benzene-1,3,5-tri­carboxyl­ato)cobalt complex, **2**, has shown excellent bifunctional catalytic activity and durability for both the oxygen-reduction reaction and oxygen-evolution reaction in alkaline media (Wang *et al.*, 2020 ▸). Unfortunately, the solvothermal synthesis required to produce the material hinders its implementation on a large scale. 
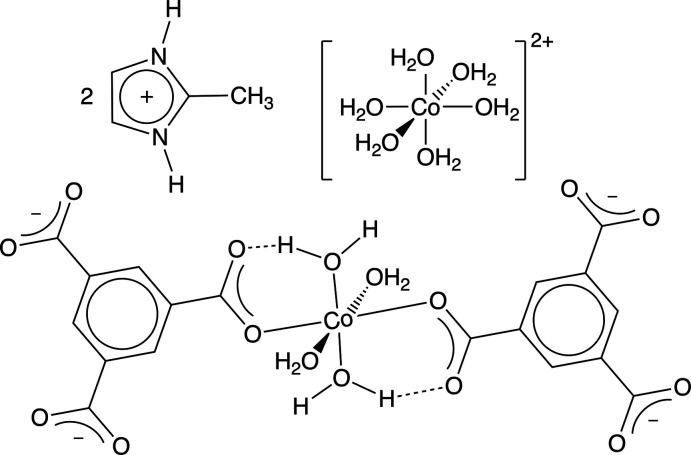


Herein, we present the synthesis and structure of a hexa­aqua­cobalt bis­(1*H*-2-methyl-imidazol-3-ium) tetra­aqua­bis­(benzene-1,3,5-tri­carboxyl­ato)cobalt complex, a related mat­erial with the imidazolium cations replaced by 2-methyl­imidazolium, which can be obtained under ambient conditions. The introduction of the methyl substituent to the C_2_ position of the imidazolium ring induces only small structural changes, when compared to **2**, and therefore, the title compound could be a promising material for ORR and OER.

## 2. Structural commentary

**Figure 1:**
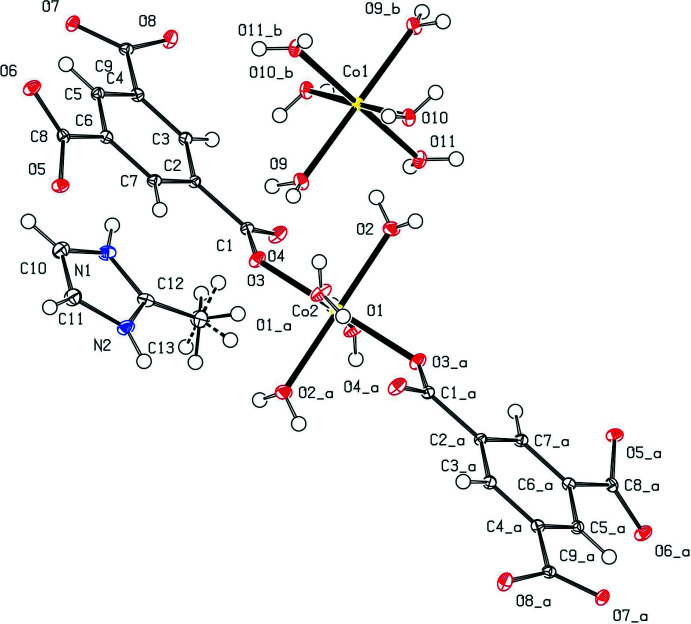
The mol­ecular structure of **1** with displacement ellipsoids drawn at the 50% probability level. Symmetry codes: (_a) 2 − *x*, −*y*, 2 − *z*; (_b) 2 − *x*, 1 − *y*, 1 − *z*.

The complete mol­ecule of **1** (Fig. 1 ▸) is generated by a crystallographic centre of symmetry. Both Co-containing ions lie about an inversion centre, and therefore only half of the coordinating ions and mol­ecules are crystallographically independent. One of the two metal centres (Co1) is coordinated by six water mol­ecules to constitute a hexa­aqua­cobalt cation, while the second (Co2) binds with four water mol­ecules and two carboxyl­ate oxygen atoms from two btc^3−^ ligands to form a [Co(H_2_O)_4_(btc)_2_]^4−^ anion. Charge neutrality of the mol­ecule is provided by the presence of two 1-*H*-2-methyl-imidazol-3-ium cations. The observed Co—O_carboxyl­ate_ bond length is 2.0835 (9) Å and the C—O_water_ bond lengths are in the range 2.0576 (9)–2.1196 (9) Å. To estimate the distortion from the ideal octa­hedral geometry, the parameters Σ (Halcrow, 2011 ▸) and Θ (Marchivie *et al.*, 2005 ▸) were calculated using the *OctaDist* program (Ketkaew *et al.*, 2021 ▸). While Σ summarizes the deviation of the *cis* O—Cu—O angles from 90°, Θ indicates the degree of twist from a perfect octa­hedron towards a trigonal prism. Both parameters are equal to zero for an ideal octa­hedron. The calculated values of the distortion parameters Σ/Θ for Co1 and Co2 are equal to 19°/62° and 11°/31°, respectively. Both parameters indicate a slight distortion of the coordination environment of both metal centres.

## 3. Supra­molecular features

**Figure 2:**
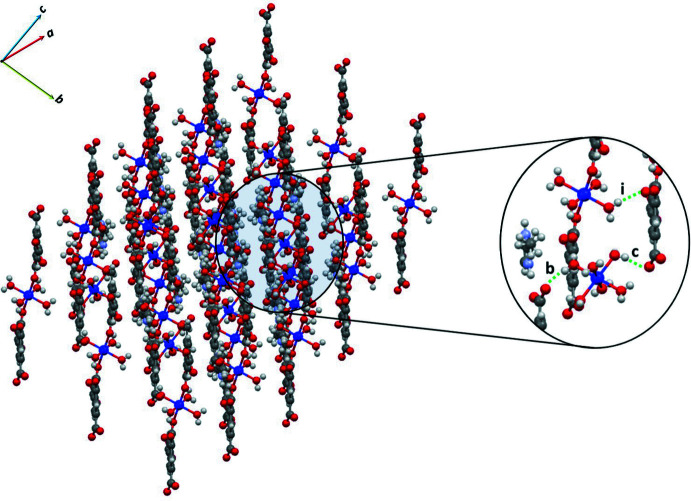
Packing diagram of **1** down the [ 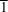 01] direction. O—H⋯O hydrogen bonds of type *b*, *c* and *i* are shown in the magnified region.

**Figure 3:**
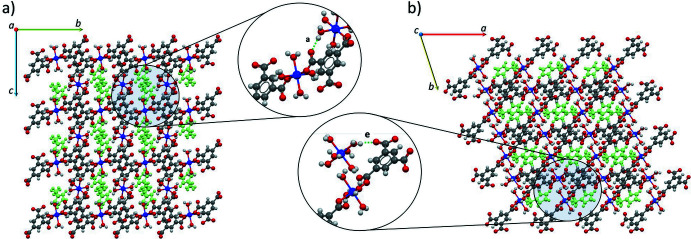
The three-dimensional supra­molecular network with one-dimensional channels along the (*a*) *a* and (*b*) *c* axes showing O—H⋯O hydrogen bonds of type *a* and *e* in the magnified area. Imidazolium ions are drawn in green for clarity.

A packing diagram of the compound as viewed down [ 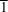 01] is shown in Fig. 2 ▸. The figure shows layers parallel to the (111) plane formed by all ions. Each ion inter­acts with others *via* hydrogen bonds of the O—H⋯O or N—H⋯O type. A summary of the hydrogen-bonding inter­actions is given in Table 1 ▸. The table demonstrates that all possible donor and acceptor groups are involved in moderate hydrogen bonds. The presence of various hydrogen bonds in **1** results in characteristic arrays that may be described by graph-set analysis (Etter *et al.*, 1990 ▸; Bernstein *et al.*, 1995 ▸). In the structure of **1**, there are 27 possible motifs involved in discrete *D* (types *a–f* and *k–l*) and inter­molecular *S* (type *h*) motifs, as well as rings *R* (types *g*, *i* and *j*) and chains *C* (types *a–f*). It is worth noting that while hydrogen bonds *b*, *c* and *i* hold the aforementioned layers together through 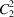 (20) and *D* arrays, other hydrogen bonds, such as type *a* and *e*, form 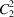 (20) arrays, which generates a three-dimensional network with channels along the *a* and *c* axes in which the imidazolium ions are located (Fig. 3 ▸).

## 4. Database survey

A search of the Cambridge Structural Database (CSD version 5.41, update of August 2020; Groom *et al.*, 2016 ▸) for hexa­aqua­cobalt and the ditrimesate tetra­aqua­cobalt moiety revealed only one hit, namely refcode: VUHQIA (imidazolium)_2_[Co(H_2_O)_6_][Co(H_2_O)_4_(btc)_2_], **2**, (Wang *et al.*, 2020 ▸). Compounds **1** and **2** crystallize in the triclinic system, space group *P* 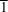 . The Co—O_carboxyl­ate_ and C—O_water_ bond lengths are similar in both complexes. The coordination polyhedra of compound **2** are slightly more distorted. The calculated values of Σ/Θ for compound **2** are equal to 21°/63° for Co1 and 10°/39° for Co2 – that is, the trigonal distortion (Θ) in **2** is higher by 1 and 8° for Co1 and Co2, respectively. The slightly different distortion of the metal centres in **2** and the introduction of the imidazolium allow for shorter hydrogen bonds with distances between 1.73 and 2.00 Å. Other complexes with a low degree of similarity to the title compound were also found, for example refcodes DOWFUS (Clegg & Holcroft, 2014 ▸), IQOZUK (Li *et al.*, 2011 ▸) and SETQOX (Wolodkiewicz *et al.*, 1996 ▸). However, these compounds are polymeric and/or incorporate a different organic ligand than btc. Additionally, none of them contain the imidazolium anion. These changes in chemical composition may provide them with totally different properties than those desired for ORR and OER, and therefore, they will not be discussed further.

## 5. Synthesis and crystallization

In a typical synthesis, H-2mIm (160 mg, 1.96 mmol), Hbtc (412, 1.96 mmol) and cobalt chloride (127 mg, 0.95mmol) were dissolved in 160 ml of a 1:1:1 mixture of deionized water, ethanol and di­methyl­formamide by stirring for 10 min at room temperature. After 5 minutes, light-pink crystals of **1** were obtained. The product was collected by filtration and washed three times with ethanol.

## 6. Refinement

Crystal data, data collection and structure refinement details are summarized in Table 2 ▸. Positions of remaining non-H atoms were found from the electron density difference maps. The positions of hydrogen atoms were refined with *U*_iso_(H) = 1.5*U*_eq_(C or N) for CH and NH groups and *U*_iso_(H) = 1.5*U*_eq_(C or O) for others. The O—H and H⋯H distances in the water mol­ecules as well as the N—H distances were restrained to be approximately equal within each type (*SHELXL* instruction SADI). The protons of the methyl group were refined as disordered over two geometrically idealized positions.

## Supplementary Material

Crystal structure: contains datablock(s) I. DOI: 10.1107/S2056989022007046/jq2016sup1.cif

Structure factors: contains datablock(s) I. DOI: 10.1107/S2056989022007046/jq2016Isup6.hkl

checkcif file. DOI: 10.1107/S2056989022007046/jq2016sup4.pdf

CCDC reference: 2129637

Additional supporting information:  crystallographic information; 3D view; checkCIF report

## Figures and Tables

**Table 1:** Hydrogen-bond geometry (Å, °)

*D*—H⋯*A*	Type	Graph-set	*D*—H	H⋯*A*	*D*⋯*A*	*D*—H⋯*A*
O9—H9*A*⋯O4	*a*	*DC* ^2^_2_(20)	0.785 (12)	1.850 (12)	2.6339 (12)	176.4 (19)
O9—H9*B*⋯O7^iv^	*b*	*DC* ^2^_2_(20)	0.775 (12)	1.957 (12)	2.7150 (13)	165.9 (18)
O10—H10*A*⋯O7^ii^	*c*	*DC* ^2^_2_(20)	0.791 (12)	2.085 (12)	2.8592 (13)	166.1 (17)
O10—H10*B*⋯O6^iii^	*d*	*DC* ^2^_2_(20)	0.793 (12)	1.891 (12)	2.6835 (12)	176.3 (18)
O11—H11*A*⋯O8^v^	*e*	*DC* ^2^_2_(20)	0.778 (12)	1.919 (13)	2.6911 (13)	171.6 (18)
O11—H11*B*⋯O5^vi^	*f*	*DC* ^2^_2_(20)	0.809 (17)	1.896 (18)	2.6989 (13)	172.0 (17)
O1—H1*A*⋯O7^i^	*g*	*C*(10) 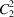 (20) 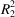 (20)	0.787 (12)	2.096 (12)	2.8789 (13)	173.5 (19)
O1—H1*B*⋯O4	*h*	*S*(6)	0.785 (12)	1.904 (13)	2.6401 (13)	155.8 (18)
O2—H2*A*⋯O5^ii^	*i*	*C*(10) 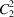 (20) 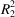 (20)	0.778 (12)	2.175 (13)	2.9371 (13)	166.5 (19)
O2—H2*B*⋯O6^iii^	*j*	*C*(10) 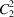 (20) 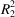 (20)	0.783 (12)	1.965 (12)	2.7458 (13)	175 (2)
N1—H1⋯O8^iv^	*k*	*DD* ^2^_2_(17)	0.913 (15)	1.811 (15)	2.7214 (13)	174.6 (16)
N2—H2⋯O5^vii^	*l*	*DD* ^2^_2_(17)	0.890 (15)	1.931 (15)	2.8206 (14)	178.0 (17)

Symmetry codes: (i) *x* + 1, *y* − 1, *z*; (ii) *x* + 1, *y*, *z*; (iii) −*x* + 1, −*y* + 1, −*z* + 2; (iv) −*x* + 1, −*y* + 1, −*z* + 1; (v) −*x* + 2, −*y* + 1, −*z* + 1; (vi) *x* + 1, *y*, *z* − 1; (vii) −*x* + 1, −*y*, −*z* + 2.

**Table 2:** Experimental details

Crystal data	
Chemical formula	(C_4_H_7_N_2_)_2_[Co(H_2_O)_6_][Co(C_9_H_3_O_6_)_2_(H_2_O)_4_]
*M*_r_	878.48
Crystal system, space group	Triclinic, *P* 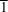
Temperature (K)	296
*a*, *b*, *c* (Å)	9.2008 (4), 9.3137 (4), 10.6470 (4)
α, β, γ (°)	86.551 (2), 79.378 (2), 72.369 (2)
*V* (Å^3^)	854.61 (6)
*Z*	1
Radiation type	Mo *K*α
μ (mm^−1^)	1.07
Crystal size (mm)	0.20 × 0.10 × 0.10

Data collection	
Diffractometer	Bruker CCD area detector
Absorption correction	Multi-scan (*SADABS*; Krause *et al.*, 2015 ▸)
*T*_min_, *T*_max_	0.699, 0.746
No. of measured, independent and observed [*I* > 2σ(*I*)] reflections	26203, 5250, 4605
*R*_int_	0.026
(sin θ/λ)_max_ (Å^−1^)	0.719

Refinement	
*R*[*F* ^2^ > 2σ(*F* ^2^)], *wR*(*F* ^2^), *S*	0.026, 0.069, 1.04
No. of reflections	5250
No. of parameters	285
No. of restraints	47
H-atom treatment	H atoms treated by a mixture of independent and constrained refinement
Δρ_max_, Δρ_min_ (e Å^−3^)	0.60, −0.42

Computer programs: *APEX2* and *SAINT* (Bruker, 2016 ▸), *SHELXT2018/2* (Sheldrick, 2018 ▸), *SHELXL2019/2* (Sheldrick, 2015 ▸) and *OLEX2* (Dolomanov *et al.*, 2009 ▸).

## supplementary crystallographic information

### Crystal data

**Table:**

(C_4_H_7_N_2_)_2_[Co(H_2_O)_6_][Co(C_9_H_3_O_6_)_2_(H_2_O)_4_]	*Z* = 1
*M**_r_* = 878.48	*F*(000) = 454
Triclinic, *P*1	*D*_x_ = 1.707 Mg m^−^^3^
*a* = 9.2008 (4) Å	Mo *K*α radiation, λ = 0.71073 Å
*b* = 9.3137 (4) Å	Cell parameters from 9913 reflections
*c* = 10.6470 (4) Å	θ = 2.3–30.6°
α = 86.551 (2)°	µ = 1.07 mm^−^^1^
β = 79.378 (2)°	*T* = 296 K
γ = 72.369 (2)°	Prism, clear light pink
*V* = 854.61 (6) Å^3^	0.20 × 0.10 × 0.10 mm

### Data collection

**Table:**

Bruker CCD area detector diffractometer	4605 reflections with *I* > 2σ(*I*)
phi and ω scans	*R*_int_ = 0.026
Absorption correction: multi-scan (SADABS; Krause *et al.*, 2015)	θ_max_ = 30.7°, θ_min_ = 2.0°
*T*_min_ = 0.699, *T*_max_ = 0.746	*h* = −13→13
26203 measured reflections	*k* = −13→12
5250 independent reflections	*l* = −15→14

### Refinement

**Table:**

Refinement on *F*^2^	Primary atom site location: difference Fourier map
Least-squares matrix: full	Secondary atom site location: dual
*R*[*F*^2^ > 2σ(*F*^2^)] = 0.026	Hydrogen site location: mixed
*wR*(*F*^2^) = 0.069	H atoms treated by a mixture of independent and constrained refinement
*S* = 1.04	*w* = 1/[σ^2^(*F*_o_^2^) + (0.0331*P*)^2^ + 0.4935*P*] where *P* = (*F*_o_^2^ + 2*F*_c_^2^)/3
5250 reflections	(Δ/σ)_max_ < 0.001
285 parameters	Δρ_max_ = 0.60 e Å^−^^3^
47 restraints	Δρ_min_ = −0.42 e Å^−^^3^

### Special details

**Table:**

Geometry. All esds (except the esd in the dihedral angle between two l.s. planes) are estimated using the full covariance matrix. The cell esds are taken into account individually in the estimation of esds in distances, angles and torsion angles; correlations between esds in cell parameters are only used when they are defined by crystal symmetry. An approximate (isotropic) treatment of cell esds is used for estimating esds involving l.s. planes.

### Fractional atomic coordinates and isotropic or equivalent isotropic displacement parameters (Å^2^)

**Table:**

	*x*	*y*	*z*	*U*_iso_*/*U*_eq_	Occ. (<1)
Co1	1.000000	0.500000	0.500000	0.00661 (6)	
Co2	1.000000	0.000000	1.000000	0.00734 (6)	
O1	1.09226 (11)	−0.04186 (11)	0.80639 (9)	0.01446 (19)	
H1A	1.128 (2)	−0.1158 (16)	0.7654 (16)	0.022*	
H1B	1.0319 (19)	0.0150 (17)	0.7699 (17)	0.022*	
O2	1.06874 (11)	0.19677 (11)	0.98183 (10)	0.01395 (18)	
H2A	1.107 (2)	0.215 (2)	1.0361 (15)	0.021*	
H2B	1.0153 (19)	0.2726 (17)	0.9590 (17)	0.021*	
O3	0.78549 (10)	0.12296 (10)	0.95554 (8)	0.01077 (17)	
O4	0.85774 (10)	0.18407 (10)	0.75266 (8)	0.01246 (18)	
O5	0.23219 (10)	0.29950 (10)	1.15378 (8)	0.01007 (16)	
O6	0.10354 (10)	0.53500 (10)	1.10793 (8)	0.01151 (17)	
O7	0.24698 (10)	0.69230 (10)	0.65394 (8)	0.00982 (16)	
O8	0.49974 (10)	0.65513 (10)	0.58167 (8)	0.01243 (18)	
O9	0.91967 (10)	0.31282 (10)	0.53041 (9)	0.01031 (17)	
H9A	0.898 (2)	0.278 (2)	0.5975 (12)	0.015*	
H9B	0.8655 (19)	0.302 (2)	0.4862 (14)	0.015*	
O10	1.09204 (10)	0.46619 (10)	0.67157 (8)	0.00996 (16)	
H10A	1.1344 (18)	0.5265 (17)	0.6797 (16)	0.015*	
H10B	1.0313 (17)	0.4689 (19)	0.7355 (13)	0.015*	
O11	1.20606 (10)	0.36656 (11)	0.40161 (9)	0.01061 (17)	
H11A	1.2898 (16)	0.368 (2)	0.4028 (16)	0.016*	
H11B	1.2048 (19)	0.350 (2)	0.3281 (17)	0.016*	
N1	0.50458 (13)	0.14060 (12)	0.61249 (10)	0.0119 (2)	
H1	0.510 (2)	0.2077 (18)	0.5469 (15)	0.014*	
N2	0.57535 (12)	−0.03974 (12)	0.74458 (10)	0.0119 (2)	
H2	0.6351 (19)	−0.1208 (18)	0.7785 (16)	0.014*	
C1	0.76058 (13)	0.19797 (13)	0.85484 (11)	0.0081 (2)	
C2	0.60272 (13)	0.31087 (13)	0.85916 (11)	0.0074 (2)	
C3	0.57120 (13)	0.41453 (13)	0.76032 (11)	0.0082 (2)	
H3	0.647875	0.413232	0.689599	0.010*	
C4	0.42546 (13)	0.52015 (13)	0.76680 (11)	0.0079 (2)	
C5	0.31078 (13)	0.52211 (13)	0.87313 (11)	0.0082 (2)	
H5	0.213899	0.593210	0.878284	0.010*	
C6	0.34094 (13)	0.41785 (13)	0.97165 (11)	0.0076 (2)	
C7	0.48664 (13)	0.31248 (13)	0.96411 (11)	0.0081 (2)	
H7	0.506641	0.242605	1.029673	0.010*	
C8	0.21698 (13)	0.41903 (13)	1.08554 (11)	0.0078 (2)	
C9	0.38899 (13)	0.63054 (13)	0.65934 (11)	0.0081 (2)	
C10	0.37595 (15)	0.14698 (15)	0.70562 (13)	0.0143 (2)	
H10	0.277987	0.216042	0.710213	0.017*	
C11	0.42065 (15)	0.03308 (15)	0.78863 (12)	0.0137 (2)	
H11	0.359239	0.008535	0.861228	0.016*	
C12	0.62411 (14)	0.02705 (14)	0.63723 (12)	0.0110 (2)	
C13	0.78039 (15)	−0.01811 (15)	0.55720 (13)	0.0156 (2)	
H13A	0.806962	−0.121788	0.533113	0.023*	0.58 (5)
H13B	0.854729	−0.005633	0.604826	0.023*	0.58 (5)
H13C	0.780728	0.043824	0.481802	0.023*	0.58 (5)
H13D	0.804580	0.069870	0.519273	0.023*	0.42 (5)
H13E	0.782325	−0.084695	0.491083	0.023*	0.42 (5)
H13F	0.855513	−0.068771	0.609385	0.023*	0.42 (5)

### Atomic displacement parameters (Å^2^)

**Table:**

	*U*^11^	*U*^22^	*U*^33^	*U*^12^	*U*^13^	*U*^23^
Co1	0.00602 (10)	0.00746 (11)	0.00589 (10)	−0.00185 (8)	−0.00054 (7)	0.00102 (7)
Co2	0.00718 (10)	0.00685 (11)	0.00737 (10)	−0.00111 (8)	−0.00184 (7)	0.00140 (8)
O1	0.0144 (4)	0.0133 (5)	0.0109 (4)	0.0036 (4)	−0.0031 (3)	−0.0002 (3)
O2	0.0155 (4)	0.0092 (4)	0.0193 (5)	−0.0037 (3)	−0.0097 (4)	0.0036 (3)
O3	0.0100 (4)	0.0115 (4)	0.0088 (4)	−0.0005 (3)	−0.0021 (3)	0.0035 (3)
O4	0.0111 (4)	0.0131 (4)	0.0089 (4)	0.0006 (3)	0.0006 (3)	0.0034 (3)
O5	0.0123 (4)	0.0081 (4)	0.0085 (4)	−0.0021 (3)	−0.0008 (3)	0.0027 (3)
O6	0.0101 (4)	0.0103 (4)	0.0105 (4)	0.0001 (3)	0.0013 (3)	0.0021 (3)
O7	0.0078 (4)	0.0110 (4)	0.0099 (4)	−0.0013 (3)	−0.0029 (3)	0.0019 (3)
O8	0.0091 (4)	0.0151 (4)	0.0116 (4)	−0.0030 (3)	−0.0012 (3)	0.0066 (3)
O9	0.0123 (4)	0.0129 (4)	0.0077 (4)	−0.0067 (3)	−0.0029 (3)	0.0038 (3)
O10	0.0089 (4)	0.0138 (4)	0.0077 (4)	−0.0047 (3)	−0.0011 (3)	0.0016 (3)
O11	0.0077 (4)	0.0144 (4)	0.0092 (4)	−0.0028 (3)	−0.0006 (3)	−0.0009 (3)
N1	0.0147 (5)	0.0096 (5)	0.0111 (5)	−0.0029 (4)	−0.0036 (4)	0.0030 (4)
N2	0.0116 (5)	0.0096 (5)	0.0127 (5)	−0.0012 (4)	−0.0017 (4)	0.0031 (4)
C1	0.0081 (5)	0.0075 (5)	0.0086 (5)	−0.0020 (4)	−0.0021 (4)	0.0009 (4)
C2	0.0073 (5)	0.0067 (5)	0.0080 (5)	−0.0016 (4)	−0.0021 (4)	0.0004 (4)
C3	0.0082 (5)	0.0083 (5)	0.0079 (5)	−0.0026 (4)	−0.0014 (4)	0.0011 (4)
C4	0.0089 (5)	0.0084 (5)	0.0068 (5)	−0.0033 (4)	−0.0020 (4)	0.0018 (4)
C5	0.0081 (5)	0.0082 (5)	0.0082 (5)	−0.0022 (4)	−0.0018 (4)	0.0005 (4)
C6	0.0084 (5)	0.0079 (5)	0.0066 (5)	−0.0028 (4)	−0.0010 (4)	0.0007 (4)
C7	0.0095 (5)	0.0073 (5)	0.0074 (5)	−0.0022 (4)	−0.0024 (4)	0.0014 (4)
C8	0.0086 (5)	0.0094 (5)	0.0067 (5)	−0.0039 (4)	−0.0027 (4)	0.0005 (4)
C9	0.0099 (5)	0.0072 (5)	0.0077 (5)	−0.0025 (4)	−0.0026 (4)	0.0003 (4)
C10	0.0121 (5)	0.0126 (6)	0.0171 (6)	−0.0020 (4)	−0.0028 (4)	0.0015 (5)
C11	0.0123 (5)	0.0136 (6)	0.0134 (6)	−0.0030 (5)	0.0000 (4)	0.0017 (4)
C12	0.0140 (5)	0.0082 (5)	0.0113 (5)	−0.0042 (4)	−0.0022 (4)	0.0005 (4)
C13	0.0148 (6)	0.0137 (6)	0.0168 (6)	−0.0046 (5)	0.0023 (5)	−0.0015 (5)

### Geometric parameters (Å, º)

**Table:**

Co1—O11	2.0576 (9)	N1—C12	1.3289 (16)
Co1—O11^i^	2.0576 (9)	N1—C10	1.3854 (16)
Co1—O9^i^	2.0772 (9)	N1—H1	0.913 (15)
Co1—O9	2.0772 (9)	N2—C12	1.3375 (15)
Co1—O10	2.1196 (9)	N2—C11	1.3832 (16)
Co1—O10^i^	2.1196 (9)	N2—H2	0.890 (15)
Co2—O3^ii^	2.0835 (9)	C1—C2	1.5078 (16)
Co2—O3	2.0835 (9)	C2—C7	1.3934 (15)
Co2—O1^ii^	2.0912 (9)	C2—C3	1.3944 (16)
Co2—O1	2.0912 (9)	C3—C4	1.3948 (16)
Co2—O2^ii^	2.1000 (9)	C3—H3	0.9300
Co2—O2	2.1000 (9)	C4—C5	1.3949 (15)
O1—H1A	0.787 (12)	C4—C9	1.5066 (16)
O1—H1B	0.785 (12)	C5—C6	1.3934 (15)
O2—H2A	0.778 (12)	C5—H5	0.9300
O2—H2B	0.783 (12)	C6—C7	1.3926 (16)
O3—C1	1.2624 (14)	C6—C8	1.5019 (15)
O4—C1	1.2601 (14)	C7—H7	0.9300
O5—C8	1.2777 (14)	C10—C11	1.3537 (17)
O6—C8	1.2526 (15)	C10—H10	0.9300
O7—C9	1.2687 (14)	C11—H11	0.9300
O8—C9	1.2580 (14)	C12—C13	1.4818 (17)
O9—H9A	0.785 (12)	C13—H13A	0.9600
O9—H9B	0.775 (12)	C13—H13B	0.9600
O10—H10A	0.791 (12)	C13—H13C	0.9600
O10—H10B	0.793 (12)	C13—H13D	0.9600
O11—H11A	0.778 (12)	C13—H13E	0.9600
O11—H11B	0.809 (17)	C13—H13F	0.9600
			
O11—Co1—O11^i^	180.0	C7—C2—C3	119.40 (10)
O11—Co1—O9^i^	90.60 (4)	C7—C2—C1	119.49 (10)
O11^i^—Co1—O9^i^	89.40 (4)	C3—C2—C1	121.11 (10)
O11—Co1—O9	89.40 (4)	C2—C3—C4	120.45 (10)
O11^i^—Co1—O9	90.60 (4)	C2—C3—H3	119.8
O9^i^—Co1—O9	180.0	C4—C3—H3	119.8
O11—Co1—O10	90.76 (4)	C3—C4—C5	119.66 (10)
O11^i^—Co1—O10	89.24 (4)	C3—C4—C9	120.93 (10)
O9^i^—Co1—O10	86.64 (3)	C5—C4—C9	119.40 (10)
O9—Co1—O10	93.36 (4)	C6—C5—C4	120.20 (11)
O11—Co1—O10^i^	89.24 (4)	C6—C5—H5	119.9
O11^i^—Co1—O10^i^	90.76 (4)	C4—C5—H5	119.9
O9^i^—Co1—O10^i^	93.36 (3)	C7—C6—C5	119.71 (10)
O9—Co1—O10^i^	86.64 (4)	C7—C6—C8	120.09 (10)
O10—Co1—O10^i^	180.0	C5—C6—C8	120.19 (10)
O3^ii^—Co2—O3	180.00 (5)	C6—C7—C2	120.56 (10)
O3^ii^—Co2—O1^ii^	90.97 (4)	C6—C7—H7	119.7
O3—Co2—O1^ii^	89.03 (4)	C2—C7—H7	119.7
O3^ii^—Co2—O1	89.03 (4)	O6—C8—O5	123.36 (11)
O3—Co2—O1	90.97 (4)	O6—C8—C6	118.94 (10)
O1^ii^—Co2—O1	180.0	O5—C8—C6	117.70 (10)
O3^ii^—Co2—O2^ii^	89.79 (4)	O8—C9—O7	124.60 (11)
O3—Co2—O2^ii^	90.21 (4)	O8—C9—C4	118.45 (10)
O1^ii^—Co2—O2^ii^	88.38 (4)	O7—C9—C4	116.95 (10)
O1—Co2—O2^ii^	91.62 (4)	C11—C10—N1	106.62 (11)
O3^ii^—Co2—O2	90.21 (4)	C11—C10—H10	126.7
O3—Co2—O2	89.79 (4)	N1—C10—H10	126.7
O1^ii^—Co2—O2	91.62 (4)	C10—C11—N2	106.72 (11)
O1—Co2—O2	88.38 (4)	C10—C11—H11	126.6
O2^ii^—Co2—O2	180.0	N2—C11—H11	126.6
Co2—O1—H1A	133.6 (14)	N1—C12—N2	107.33 (11)
Co2—O1—H1B	104.8 (13)	N1—C12—C13	125.58 (11)
H1A—O1—H1B	107.5 (16)	N2—C12—C13	127.07 (11)
Co2—O2—H2A	117.6 (14)	C12—C13—H13A	109.5
Co2—O2—H2B	121.0 (13)	C12—C13—H13B	109.5
H2A—O2—H2B	108.3 (16)	H13A—C13—H13B	109.5
C1—O3—Co2	126.90 (8)	C12—C13—H13C	109.5
Co1—O9—H9A	125.1 (13)	H13A—C13—H13C	109.5
Co1—O9—H9B	117.8 (13)	H13B—C13—H13C	109.5
H9A—O9—H9B	108.5 (16)	C12—C13—H13D	109.5
Co1—O10—H10A	111.8 (12)	H13A—C13—H13D	135.3
Co1—O10—H10B	115.7 (13)	H13B—C13—H13D	76.7
H10A—O10—H10B	106.0 (15)	H13C—C13—H13D	35.5
Co1—O11—H11A	128.6 (13)	C12—C13—H13E	109.5
Co1—O11—H11B	114.9 (12)	H13A—C13—H13E	35.5
H11A—O11—H11B	105.6 (16)	H13B—C13—H13E	135.3
C12—N1—C10	109.77 (10)	H13C—C13—H13E	76.7
C12—N1—H1	124.4 (11)	H13D—C13—H13E	109.5
C10—N1—H1	125.7 (11)	C12—C13—H13F	109.5
C12—N2—C11	109.55 (11)	H13A—C13—H13F	76.7
C12—N2—H2	123.4 (11)	H13B—C13—H13F	35.5
C11—N2—H2	127.0 (11)	H13C—C13—H13F	135.3
O4—C1—O3	124.51 (11)	H13D—C13—H13F	109.5
O4—C1—C2	118.73 (10)	H13E—C13—H13F	109.5
O3—C1—C2	116.76 (10)		
			
Co2—O3—C1—O4	16.76 (18)	C1—C2—C7—C6	178.56 (11)
Co2—O3—C1—C2	−163.49 (8)	C7—C6—C8—O6	161.30 (11)
O4—C1—C2—C7	170.61 (11)	C5—C6—C8—O6	−18.68 (17)
O3—C1—C2—C7	−9.15 (17)	C7—C6—C8—O5	−19.76 (17)
O4—C1—C2—C3	−9.89 (17)	C5—C6—C8—O5	160.27 (11)
O3—C1—C2—C3	170.35 (11)	C3—C4—C9—O8	−19.45 (17)
C7—C2—C3—C4	0.72 (18)	C5—C4—C9—O8	161.70 (11)
C1—C2—C3—C4	−178.78 (11)	C3—C4—C9—O7	160.47 (11)
C2—C3—C4—C5	0.16 (18)	C5—C4—C9—O7	−18.38 (17)
C2—C3—C4—C9	−178.68 (11)	C12—N1—C10—C11	−0.03 (15)
C3—C4—C5—C6	−0.83 (18)	N1—C10—C11—N2	0.19 (15)
C9—C4—C5—C6	178.03 (11)	C12—N2—C11—C10	−0.29 (15)
C4—C5—C6—C7	0.61 (18)	C10—N1—C12—N2	−0.15 (15)
C4—C5—C6—C8	−179.42 (11)	C10—N1—C12—C13	178.19 (12)
C5—C6—C7—C2	0.29 (18)	C11—N2—C12—N1	0.27 (15)
C8—C6—C7—C2	−179.69 (11)	C11—N2—C12—C13	−178.04 (13)
C3—C2—C7—C6	−0.95 (18)		

Symmetry codes: (i) −*x*+2, −*y*+1, −*z*+1; (ii) −*x*+2, −*y*, −*z*+2.

### Hydrogen-bond geometry (Å, º)

**Table:**

*D*—H···*A*	*D*—H	H···*A*	*D*···*A*	*D*—H···*A*
O1—H1*A*···O7^iii^	0.79 (1)	2.10 (1)	2.8789 (13)	174 (2)
O1—H1*B*···O4	0.79 (1)	1.90 (1)	2.6401 (13)	156 (2)
O2—H2*A*···O5^iv^	0.78 (1)	2.17 (1)	2.9371 (13)	167 (2)
O2—H2*B*···O6^v^	0.78 (1)	1.97 (1)	2.7458 (13)	175 (2)
O9—H9*A*···O4	0.79 (1)	1.85 (1)	2.6339 (12)	176 (2)
O9—H9*B*···O7^vi^	0.78 (1)	1.96 (1)	2.7150 (13)	166 (2)
O10—H10*A*···O7^iv^	0.79 (1)	2.09 (1)	2.8592 (13)	166 (2)
O10—H10*B*···O6^v^	0.79 (1)	1.89 (1)	2.6835 (12)	176 (2)
O11—H11*A*···O8^i^	0.78 (1)	1.92 (1)	2.6911 (13)	172 (2)
O11—H11*B*···O5^vii^	0.81 (2)	1.90 (2)	2.6989 (13)	172 (2)
N1—H1···O8^vi^	0.91 (2)	1.81 (2)	2.7214 (13)	175 (2)
N2—H2···O5^viii^	0.89 (2)	1.93 (2)	2.8206 (14)	178 (2)

Symmetry codes: (i) −*x*+2, −*y*+1, −*z*+1; (iii) *x*+1, *y*−1, *z*; (iv) *x*+1, *y*, *z*; (v) −*x*+1, −*y*+1, −*z*+2; (vi) −*x*+1, −*y*+1, −*z*+1; (vii) *x*+1, *y*, *z*−1; (viii) −*x*+1, −*y*, −*z*+2.
