# Supplementary material for: Synthesis and structure of hexa­aqua­cobalt bis­(2-methyl-1H-imidazol-3-ium) tetra­aqua­bis­(benzene-1,3,5-tri­carboxyl­ato-κO)cobalt
Source: Acta Crystallogr E Crystallogr Commun. 2022 Jul 19;78(Pt 8):814–7. doi: 10.1107/S2056989022007046 (PMC9361360; doi:10.1107/S2056989022007046)

checkCIF/PLATON report

Structure factors have been supplied for datablock(s) I

THIS REPORT IS FOR GUIDANCE ONLY. IF USED AS PART OF A REVIEW PROCEDURE FOR PUBLICATION, IT SHOULD NOT REPLACE THE EXPERTISE OF AN EXPERIENCED CRYSTALLOGRAPHIC REFEREE.

No syntax errors found. CIF dictionary Interpreting this report

Datablock: I

Bond precision:	C-C = 0.0017 A		Wavelength=0.71073
Cell:	a=9.2008(4)	b=9.3137(4)	c=10.6470(4)
	alpha=86.551(2)	beta=79.378(2)	gamma=72.369(2)
Temperature:	296 K		

	Calculated	Reported
Volume	854.61(6)	854.61(6)
Space group	P -1	P -1
Hall group	-P 1	-P 1
Moiety formula	C18 H14 Co O16, Co H12 O6, 2(C4 H7 N2)	C18 H14 Co O16, Co H12 O6, 2(C4 H7 N2)
Sum formula	C26 H40 Co2 N4 O22	C26 H40 Co2 N4 O22
Mr	878.48	878.48
Dx,g cm-3	1.707	1.707
Z	1	1
Mu (mm-1)	1.069	1.069
F000	454.0	454.0
F000'	454.97	
h,k,lmax	13,13,15	13,13,15
Nref	5312	5250
Tmin,Tmax	0.880,0.899	0.699,0.746
Tmin'	0.808	

Correction method= # Reported T Limits: Tmin=0.699 Tmax=0.746
AbsCorr = MULTI-SCAN

Data completeness= 0.988 Theta(max)= 30.721

R(reflections)= 0.0263(4605) wR2(reflections)= 0.0693(5250)

S = 1.036 Npar= 285

The following ALERTS were generated. Each ALERT has the format
test-name_ALERT_alert-type_alert-level.
Click on the hyperlinks for more details of the test.

Alert level C

```
PLAT911_ALERT_3_C Missing FCF Refl Between Thmin & STh/L=    0.600          8 Report
```

Alert level G

```
PLAT002_ALERT_2_G Number of Distance or Angle Restraints on AtSite         19 Note
PLAT154_ALERT_1_G The s.u.'s on the Cell Angles are Equal ..(Note)      0.002 Degree
PLAT176_ALERT_4_G The CIF-Embedded .res File Contains SADI Records          3 Report
PLAT794_ALERT_5_G Tentative Bond Valency for Co2       (II)      .       2.04 Info
PLAT860_ALERT_3_G Number of Least-Squares Restraints .............         47 Note
PLAT912_ALERT_4_G Missing # of FCF Reflections Above STh/L=  0.600         54 Note
PLAT978_ALERT_2_G Number C-C Bonds with Positive Residual Density.          8 Info
```

0 **ALERT level A** = Most likely a serious problem - resolve or explain
0 **ALERT level B** = A potentially serious problem, consider carefully
1 **ALERT level C** = Check. Ensure it is not caused by an omission or oversight
7 **ALERT level G** = General information/check it is not something unexpected

1 ALERT type 1 CIF construction/syntax error, inconsistent or missing data
2 ALERT type 2 Indicator that the structure model may be wrong or deficient
2 ALERT type 3 Indicator that the structure quality may be low
2 ALERT type 4 Improvement, methodology, query or suggestion
1 ALERT type 5 Informative message, check

Publication of your CIF

You should attempt to resolve as many as possible of the alerts in all categories. Often the minor alerts point to easily fixed oversights, errors and omissions in your CIF or refinement strategy, so attention to these fine details can be worthwhile. In order to resolve some of the more serious problems it may be necessary to carry out additional measurements or structure refinements. However, the nature of your study may justify the reported deviations from journal submission requirements and the more serious of these should be commented upon in the discussion or experimental section of a paper or in the "special_details" fields of the CIF. *checkCIF* was carefully designed to identify outliers and unusual parameters, but every test has its limitations and alerts that are not important in a particular case may appear. Conversely, the absence of alerts does not guarantee there are no aspects of the results needing attention. It is up to the individual to critically assess their own results and, if necessary, seek expert advice.

If you wish to submit your CIF for publication in Acta Crystallographica Section C or E, you should upload your CIF via the web. If you wish to submit your CIF for publication in IUCrData you should upload your CIF via the web. If your CIF is to form part of a submission to another IUCr journal, you will be asked, either during electronic submission or by the Co-editor handling your paper, to upload your CIF via our web site.

PLATON version of 18/05/2022; check.def file version of 19/01/2022

Datablock I - ellipsoid plot

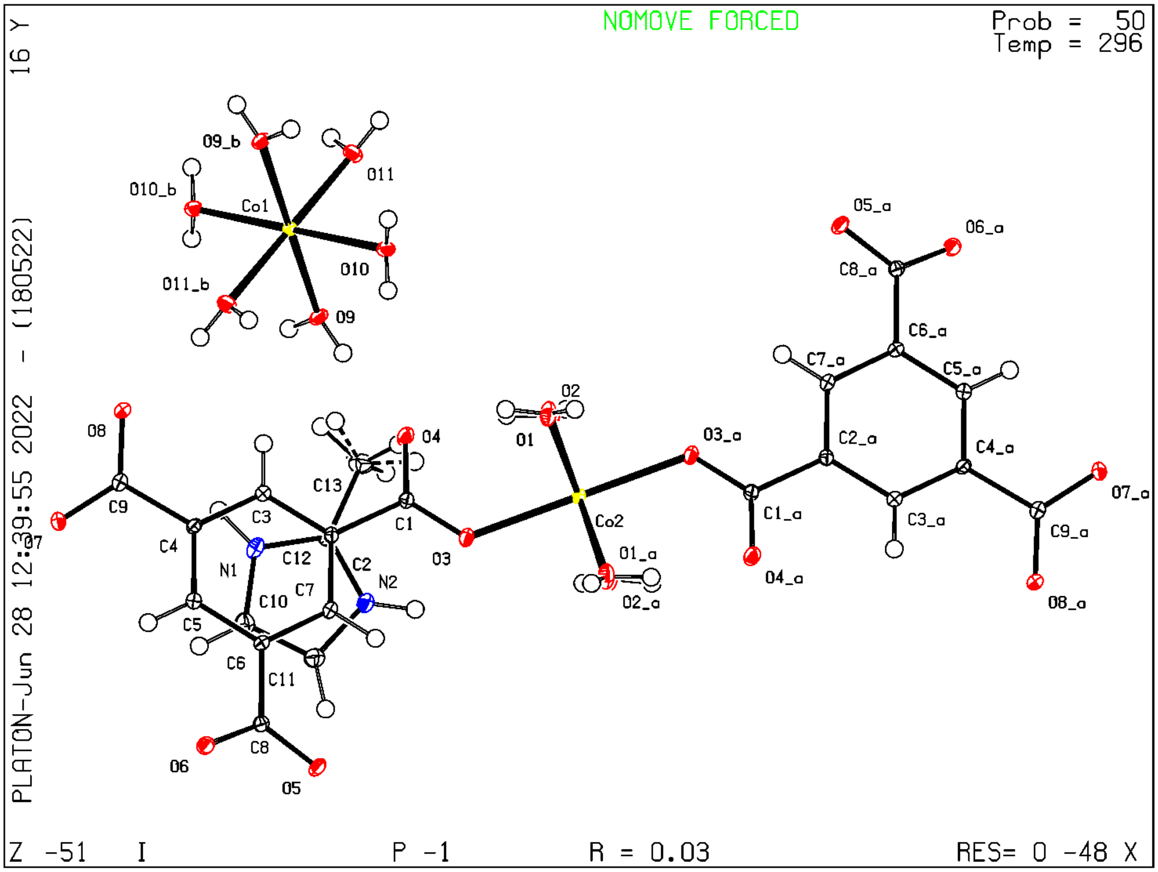

Supplement: Supplementary file 3 [file e-78-00814-sup4.pdf]
